# Breastfeeding: what changed after a decade?^1^

**DOI:** 10.1590/1518-8345.1858.2941

**Published:** 2017-10-30

**Authors:** Áurea Tamami Minagawa Toryiama, Elizabeth Fujimori, Claudia Nery Teixeira Palombo, Luciane Simões Duarte, Ana Luiza Vilela Borges, Christiane Borges do Nascimento Chofakian

**Affiliations:** 2 PhD, Professor, Escola de Enfermagem, Universidade de São Paulo, São Paulo, SP, Brazil.; 3 PhD, Associate Professor, Escola de Enfermagem, Universidade de São Paulo, São Paulo, SP, Brazil.; 4 Doctoral student, Escola de Enfermagem, Universidade de São Paulo, São Paulo, SP, Brazil. Scholarship holder at Coordenação de Aperfeiçoamento de Pessoal de Nível Superior (CAPES), Brazil.

**Keywords:** Breast Feeding, Child Health, Primary Care Nursing, Primary Health Care, Epidemiologic Factors, Epidemiology, Cross-Sectional Studies

## Abstract

**Objective:**

to analyze the changes in prevalence, median duration and correlates of breastfeeding in a small city in São Paulo state, Brazil.

**Method:**

analysis of two cross-sectional studies, conducted at intervals of one decade, with 261 and 302 children younger than two years, respectively. We used Kaplan-Meier survival analysis for calculation of the median duration of breastfeeding, and Cox regression for correlates analysis, with significance level of 5%.

**Results:**

an increase of 33.4% in the prevalence of exclusive breastfeeding and 20.9% in breastfeeding was identified. Regarding the latter, the median duration increased from 7.2 to 12 months. In the most recent study, the median duration was lower in first-born children who used pacifiers, and it was not associated with breastfeeding incentive actions.

**Conclusions:**

advances in the prevalence and duration of breastfeeding were observed during the 10 year-period, however, pacifier use still remains associated to a shorter median duration of breastfeeding. Our findings contribute to highlighting the need for intensification of nursing actions in the promotion of breastfeeding, and discouragement regarding the use of pacifiers.

## Introduction

The practice of breastfeeding is associated with good health conditions for children, with a primary effect of reducing the incidence and severity of the most prevalent diseases in childhood, as well as infant mortality^1^ ^-^ ^2^. Likewise, this practice has demonstrated positive effects on intelligence quotient, school performance, and income in adulthood, which translates into advantages for families and society^3^ ^-^ ^4^.

Despite the numerous benefits, the prevalence of exclusive breastfeeding for the first six months of life has yet to reach 40% of children worldwide^4^. This is also the case in Brazil, where the prevalence of exclusive breastfeeding in children under six months remains at 41%; this is higher than the 3% observed in the 1980s, which is certainly a consequence of the investment in public policies to promote breastfeeding that resulted in considerable improvements in the indicators in the country. The median duration of breastfeeding also increased from 2.5 months in 1975 to 11.3 months in 2008^5^, but remains far from 20 months, which is considered a satisfactory level^6^.

The advances in breastfeeding indicators show some heterogeneous behaviors among Brazilian capitals and regions. The prevalence of exclusive breastfeeding in children under six months varies from 27% in Cuiabá (Mid-west region), to 56% in Belém (North region), with a median duration of 0.7 to 88.8 days, respectively. The duration of breastfeeding, in turn, ranges from 293 days (3.1 months), in São Paulo (Southwest region, to 601 days (20 months), in Macapá (North region)^7^.

Such diverse behavior reinforces the need for local research to evaluate and monitor regional indicators and determinants, as the establishment and maintenance of breastfeeding are influenced by the socioeconomic, cultural, family, maternal, and child characteristics of each population, such as: family income, age, level of education, marital status, maternal work, infant sex, birth weight, birth sequence, and pacifier use^8^ ^-^ ^10^. In addition, the practice of breastfeeding is also determined by health professional practices to promote breastfeeding during prenatal, maternity, and child care periods^11^.

A study developed in the beginning of the 2000s, in a small city in the State of São Paulo, Brazil, showed a prevalence of breastfeeding in 41% of infants under 24 months, and exclusive breastfeeding in 13% of those under six months, with a median duration of 7.2 months of breastfeeding and only 28 days of exclusive breastfeeding. The correlates of lower median breastfeeding were: lactation, first order of birth, and pacifier use^12^. In view of the implementation of public policies to promote, protect, and support breastfeeding and the expansion of primary health care in the last decade in the country, as well as the conduct of a new study on health and nutrition conditions in the same city, we concluded it would be pertinent and timely to analyze the changes in prevalence, median duration, and correlates of breastfeeding between 2001 and 2013, in this small city of São Paulo state, which was the objective in this study.

The study was conducted based on the hypothesis that the prevalence and median duration of breastfeeding increased during the period under review; the correlates of the median duration of breastfeeding did not change; and, finally, the actions to promote breastfeeding (counseling on breastfeeding in prenatal and childbirth periods, breastfeeding in the first hour of the newborn’s life, exclusive breastfeeding in the maternity unit, childcare follow-up, and breastfeeding counseling in child care consultations) had a positive effect on the median duration of breastfeeding more than ten years later, in 2013.

## Methods

This is a cross-sectional study, conducted during 2013, in a small city in the State of São Paulo, Brazil, that compares data to another methodologically similar study conducted in in the same city, in 2001^12^.

In 2001, the city population was about 26 thousand inhabitants, and the local health care system consisted of a health center, seven Primary Health Care Facilities (PHCF), a Psychosocial Care Center (PCC) and a small size municipal hospital (50 beds). In 2013, the population increased to 48 thousand inhabitants, and the health system is now composed of 12 BHUs, three outpatient clinics, and has maintained the PCC and the municipal hospital^13^.

The first population-based study was conducted with a representative sample of 261 children under two years of age^12^. The second study, performed in 2013, was part of a larger project approved by the Research Ethics Committee (Process nº 193.468), which evaluated the health and nutrition of children less than three years of age enrolled in the PHCFs, in a representative sample proportional to the number of children enrolled in every PHCF. The sample calculation, performed with the Epi-info 6.04 software, indicated a sample of 350 children, with 95% confidence level and a 5% margin of error. Inclusion criteria were: child enrolled in one of the 12 PHCFs, and attendance at the health service with the mother during the period of data collection. Among the 399 mothers invited, 35 refused, one did not meet the inclusion criteria, and five were excluded (three twins, one adopted, and one with neurological disease). Of the 358 children from zero to three years old who composed the sample, 302 children under two years of age (84.4%) were analyzed in this study, and compared with the previous study.

The data collection was performed from February to April of 2013, by 11 nurses and one undergraduate nursing student, all of whom were duly qualified. The mothers were interviewed in the PHCFs, with a pre-tested questionnaire, with information on characteristics of the family (family income per capita, father’s level of education), mother (age, level of education, marital status, working), children (age, sex, birth weight, birth order, pacifier use), and actions to promote breastfeeding (guidelines in prenatal and childbirth periods, breastfeeding within the first hour after birth, breastfeeding in the maternity unit, childcare follow-up, and counseling on breastfeeding in the child-care consultations). From this last block, only information on the guidelines on breastfeeding in the prenatal and childbirth periods was similarly obtained in the 2001 study. Also in 2001, the use of the bottle was associated with a shorter duration of breastfeeding; however, we decided not to use this variable, given its consecrated and undisputed relationship with weaning.

The data were uploaded into the database, developed with Epi-info software, version 6.04, with duplicate data entry, to verify consistency; the statistical analyses were processed with the Statistical Package for the Social Sciences (SPSS) software, version 17. The dependent variables were the median duration and prevalence of exclusive breastfeeding, and breastfeeding estimated for the age groups recommended by the World Health Organization (WHO): less than four and less than six months for exclusive breastfeeding and breastfeeding, in addition to six to nine, 12 - 15 - 20 - 24 and 0 - 24 months for breastfeeding. Exclusive breastfeeding was defined as a situation in which the child received exclusively breast milk, without addition of water and/or other liquid; breastfeeding was defined as a situation in which the child received breast milk along with any other type of feeding^14^.

The median duration of exclusive breastfeeding and breastfeeding was verified using the Kaplan-Meier survival analysis. This technique enables the analysis of the time elapsed until the occurrence of a certain event, which, in this study, referred to the time from breastfeeding to weaning (definitive interruption of breastfeeding). This technique has been widely used in other studies on breastfeeding, as it gives the advantage of analyzing both information from weaned and still breastfed children at the time of the interview^15^ ^-^ ^16^. For the child who still received breast milk at the time of the interview (censored child), the time of breastfeeding referred to the current age at the interview. To evaluate the difference between breastfeeding medians in the univariate analysis, the log-rank test was used.

The identification of correlates of duration of breastfeeding was performed using multiple Cox analysis (Cox proportional hazards models). The variables were selected according to the hypotheses in the study, and two models were developed. In Model 1, only those variables that were statistically associated with the duration of breastfeeding in 2001 (birth order and pacifier use) were considered as independent variables. Model 2 was developed considering also the possible effect of the variables related to breastfeeding incentive actions not investigated in 2001 (counseling on breastfeeding in prenatal and childbirth periods, breastfeeding within the first hour of life, exclusive breastfeeding in the maternity unit, childcare and guidance on childcare in childcare appointments). Maternal variables classically associated with duration of breastfeeding were considered for model fit: marital status and maternal level of education^8^ ^,^ ^17^. Both models were developed by inserting the variables simultaneously. In the Cox regression model, the measure of association in Hazard Ratio is similar to relative risk, and indicates the likelihood of a subject who did not have the event, having it at that time. The significance level of 5% was considered statistically significant in the final model. The Schoenfeld test was used to verify the adequacy of the Cox models, and all variables met the risk proportionality assumptions.

## Results

Table 1 shows the changes in the profile of the sample studied in 2013 (n=302), in relation to the population of the 2001 study (n=261)^12^. A statistically higher percentage of families with per capita income greater than or equal to 1.8 minimum wages was found, along with a reduction in the proportion of mothers with three or more years of education, and an increase in working mothers (p<0.001). There was a 20% increase in guidance on breastfeeding in prenatal and childbirth periods (p<0.001), and a reduction in the pacifier use (p=0.05).

**Table 1:** Family, maternal, infant, and breastfeeding incentive actions presented in the 2013 study, percentage difference, and p-values in relation to the 2001 study. A small city, São Paulo state, Brazil, 2013

**Characteristics**		**2013 (%)**	**Percent difference between 2013 and 2001, and p-value**	
			**(%)**	p*
**Family** ^**†**^				
	**Family income per capita: ≥1.8 minimum wages**	**95.7**	**+13.3**	**<0.001**
	**Father’s level of education: ≥3 years of study**	**80.5**	**+3.5**	**0.157**
**Maternal**				
	**Age: >20 years**	**82.1**	**-2.6**	**0.707**
	**Marital status: with partner**	**81.5**	**-5.5**	**0.316**
	**Level of education: ≥3 years of study**	**67.2**	**-22.4**	**<0.001**
	**Working: yes**	**61.9**	**+33.5**	**<0.001**
**Children**				
	**Sex: male**	**57.6**	**+3.6**	**0.392**
	**Birth weight: ≥2500grams**	**89.1**	**-3.2**	**0.418**
	**Birth order:**			**0.210**
	**1** ^**st**^ **child**	**45.0**	**+7.1**	
	**2** ^**nd**^ **child**	**29.5**	**-5.4**	
	**3** ^**rd**^ **child or more**	**25.5**	**-1.7**	
	**Pacifier use: yes**	**45.0**	**-8.2**	**0.052**
**Actions to Promote breastfeeding** ^**‡**^				
	**Counseling on breastfeeding** ^**‡**^ **in prenatal and childbirth periods: yes**	**70.2**	**+20.0**	**<0.001**
	**Breastfeeding** ^**‡**^ **in the first hour of birth: yes** ^**§**^	**22.2**	**-**	**-**
	**Exclusive breastfeeding** ^**‡**^ **in the maternity unit: yes** ^**§**^	**24.2**		
	**Follow-up in childcare: yes**	**74.8**	**-**	**-**
	**Counseling on breastfeeding** ^**‡**^ **during childcare appointments: yes** ^**§**^	**23.2**	**-**	**-**

*chi-square test; †no information for the entire sample; ‡breastfeeding; §Data investigated only in the 2013 study

Between 2001 and 2013, the prevalence of exclusive breastfeeding increased significantly in the city (p<0.05): we observed a 40.8% increase in children under four months and a 33.4% increase in children under six months, with prevalence of 58.6 and 46.1%, respectively, in 2013.

The prevalence of breastfeeding in the city was 61.9%; an increase of 20.9% (p<0.001) during the period from 2001 to 2013, especially among the age group from six to nine months, with a prevalence of 68.4% in 2013; a growth of 53.5% in the period, in the 12-to 15-month age group, with a prevalence of 42.9% in 2013, an addition of 27.7% in the period (Table 2).

**Table 2:** Prevalence of breastfeeding and confidence interval, according to the age groups presented in the 2013 study, percent differences in prevalence between 2013 and 2001, and p-values. A small city, São Paulo state, Brazil, 2013

**Age group (month)**		**Prevalence in 2013 (%)**	**(CI 95%)**	**Percent differences in prevalence of breastfeeding (2013-2001), and p-value**	
				**%**	p*
**Exclusive breastfeeding** ^**†**^					
	**<4**	**58.6**	**(48.3-69.0)**	**+40.8**	**0.003**
	**<6**	**46.1**	**(37.5-54.7)**	**+33.4**	**<0.001**
**Breastfeeding** ^**‡**^					
	**<4**	**88.5**	**(81.8-95.2)**	**+10.7**	**0.103**
	**<6**	**82.0**	**(75.4-88.7)**	**+10.2**	**0.094**
	**6-9**	**68.4**	**(56.3-80.5)**	**+53.5**	**<0.001**
	**12-15**	**42.9**	**(27.9-57.8)**	**+27.7**	**0.090**
	**20-24**	**27.0**	**(12.7-41.3)**	**-4.6**	**0.665**
	**0-24**	**61.9**	**(56.3-67.4)**	**+20.9**	**<0.001**

*Chi-square test; †Exclusive breastfeeding; ‡Breastfeeding

**Figure 1:**
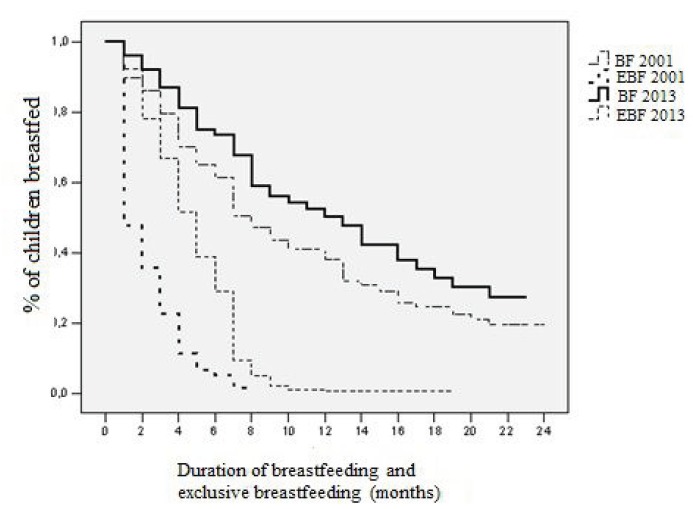
Median duration of exclusive breastfeeding and breastfeeding in 2001 and 2013. Small city, São Paulo state, Brazil, 2013.

Figure 1 shows a higher proportion of children breastfed in 2013, both in exclusive breastfeeding and breastfeeding categories. The median duration of exclusive breastfeeding was four months in 2013, and 28 days in 2001; while the duration of breastfeeding changed to 12 months in 2013, compared to 7.2 months in 2001 - an increase of 4.8 months in the period.

In the univariate analysis, the variables statistically associated with the median duration of breastfeeding, in 2013, were: marital status, birth order, and pacifier use (Table 3). The median duration of breastfeeding was lower in children whose mothers has no partners, first born infants, and those who used pacifiers. In the analysis of the difference in the median duration of breastfeeding between 2001 and 2013, according to family, maternal, and children characteristics, and breastfeeding incentive actions, the duration of breastfeeding increased near five months for many of the categories analyzed, but decreased or increased less in children with parents with less than three years of education, mothers without partners, and those under 20 years old, first or second children, children who used pacifiers, and in situations in which there was no prenatal and childbirth counseling. The median duration of breastfeeding was seven months in children who used pacifiers.

**Table 3:** Median duration of breastfeeding in 2013, and difference between 2001 and 2013, according to family, maternal and infant characteristics, and actions to promote breastfeeding. A small city, São Paulo state, Brazil, 2013

**Characteristics**			**Median duration of breastfeeding (in months)**	p-value*	**Difference in breastfeeding duration (2013-2001) (in months)**
**Family** ^**†**^					
	**Family income per capita (minimum wage)**			**0.457**	
		**<1.8**	**8**		**1.2**
		**>1.8**	**10**		**1.2**
	**Father’s level of education (years of study)**			**0.292**	
		**<3**	**17**		**-7**
		**>3**	**13**		**5.4**
**Maternal characteristics**					
	**Age (years)**			**0.093**	
		**<20**	**7**		**-3.8**
		**>20**	**13**		**5.5**
	**Marital status**			**0.004**	
		**with partner**	**7**		**2.5**
		**without partner**	**13**		**5.0**
	**Level of education (years of study)**			**0.171**	
		**<3**	**12**		**-0.6**
		**>3**	**12**		**4.5**
	**Working**			**0.474**	
		**No**	**13**		**5.1**
		**Yes**	**11**		**4.9**
**Children’s characteristics**					
	**Sex:**			**0.847**	
		**Male**	**13**		**6.1**
		**Female**	**10**		**2.2**
	**Birth weight (grams)**			**0.764**	
		**<2500**	**12**		**4.7**
		**≥2500**	**13**		**5.4**
	**Birth order**			**0.058**	
		**1** ^**st**^ **child**	**9**		**2.9**
		**2** ^**nd**^ **child**	**11**		**2.7**
		**3** ^**rd**^ **child or more**	**17**		**6.8**
	**Pacifier use**			**<0.001**	
		**No**	**7**		**2.4**
		**Yes** ^**‡**^	**-**		**-**
**Actions to Promote breastfeeding**					
	**Counseling on breastfeeding in prenatal and childbirth periods**			**0.400**	
		**No**	**11**		**3.2**
		**Yes**	**12**		**4.7**
	**Breastfeeding within the first hour of birth** ^**§**^			**0.098**	
		**No**	**8**		**-**
		**Yes**	**12**		**-**
	**Exclusive breastfeeding in the maternity unit** ^**§**^			**0.069**	
		**No**	**11**		**-**
		**Yes**	**12**		**-**
	**Follow-up in childcare** ^**§**^			**0.854**	
		**No**	**12**		**-**
		**Yes**	**13**		**-**
	**Counseling on breastfeeding during childcare appointments** ^**§**^			**0.656**	
		**No**	**10**		**-**
		**Yes**	**13**		**-**

*Log-rank test (significance test to compare groups in univariate survival analysis);†without information for the entire sample; ‡it was not possible to estimate, because the majority of children who did not use pacifiers were still breastfeeding; §data investigated only in the 2013 study

The Cox multiple regression Model 1 showed that pacifier use and birth order remained as correlates of the median duration of breastfeeding in 2013: first born infants were more likely to weaning compared to those who were third-order of birth, and infants using pacifier had a nearly five-fold increased hazard for weaning (p <0.05). In addition to the variables analyzed in Model 1, the variables related to the actions to promote breastfeeding were included in Model 2. Only the use of pacifiers and being first born remained as correlates for weaning, without statistical association with actions to promote breastfeeding (Table 4).

**Table 4:** Cox-Hazard Ratio multiple regression and statistical significance for association with weaning. Model 1: Statistically significant variables in 2001. Model 2: Statistically significant variables in 2001, and actions to promote breastfeeding in 2013. A small city, São Paulo state, Brazil, 2013

Variables*		**Model 1** ^**†**^				**Model 2** ^**†**^		
		**HR** ^**‡**^	**(CI 95%)**	**p value** ^**§**^		**HR** ^**‡**^	**(VI 95%)**	**p value** ^**§**^
**Pacifier use**								
	**No**	**1**				**1**		
	**Yes**	**4.886**	**(3.08-7.74)**	**<0.001**		**5.604**	**(3.38-9.30)**	**<0.001**
**Birth order**								
	**1** ^**st**^ **child**	**1.652**	**(1.02-2.68)**	**0.042**		**1.737**	**(1.01-2.98)**	**0.044**
	**2** ^**nd**^ **child**	**1.129**	**(0.65-1.97)**	**0.667**		**1.182**	**(0.65-2.16)**	**0.587**
	**3** ^**rd**^ **child or more**	**1**				**1**		
**Counseling on breastfeeding in prenatal and childbirth periods**								
	**No**					**1**		
	**Yes**					**1.134**	**(0.75-1.72)**	**0.554**
**Breastfeeding within the first hour of birth** ^**||**^								
	**No**					**1**		
	**Yes**					**1.070**	**(0.65-1.76)**	**0.788**
**Exclusive breastfeeding in the maternity unit** ^**||**^								
	**No**					**1**		
	**Yes**					**0.963**	**(0.58-1.60)**	**0.885**
**Follow-up in childcare** ^**||**^								
	**No**					**1**		
	**Yes**					**0.941**	**(0.45-1.95)**	**0.870**
**Counseling on breastfeeding during childcare appointments** ^**||**^								
	**No**					**1**		
	**Yes**					**1.231**	**(0.74-2.05)**	**0.425**

*Schoenfeld test performed for all variables, confirming that the risks were proportional; †adjusted for: marital status and maternal education; ‡HR = Hazard Ratio; §Multiple Cox analysis; ||Data investigated only in the 2013 study

## Discussion

Our findings corroborate the findings in national surveys^18^ ^-^ ^19^ and trend analysis of the breastfeeding performed in the last decade^7^, which shows an increase in the prevalence and median duration of breastfeeding, and also confirms the hypothesis of this investigation.

The prevalence of exclusive breastfeeding among children under four and six months of age increased significantly in the city that is the scenario of our study between 2001 and 2013. In 2013, the prevalence of exclusive breastfeeding in children under six months of age (46.1%) was higher than in 2008, either considering the city of São Paulo (39.1%) or the Southeast Region (39.4%)^19^. Even so, this prevalence is still considered poor by the WHO, which classifies as good only a prevalence that reaches a rate of 50%^6^.

On the other hand, the practice of breastfeeding not only increased from 2001 to 2013, but also was prolonged; a more expressive increase of breastfeeding was found among children from six to nine months and from 12 to 15 months, the last age group recommended by WHO^14^ ^)^ as an indicator of breastfeeding continuity. Despite the increase, the percentages found are still slightly lower than the prevalence of 87.6% of breastfeeding in children under six months, and of 47.5% in the range of 12 to 15 months, verified in Brazil in 2006^18^.

The increase in the median duration of breastfeeding, that reached 12 months in 2013, can be considered an important advance when analyzed *in loco*, however, as in 26 out of the 27 Brazilian capitals in 2008^14^, the median found here is still classified as very poor, according to WHO parameters: very poor for median 0 to 17 months; bad, from 18 to 20 months; good, from 21 to 22 months; and, finally, very good, from 23 to 24 months^6^. Even so, breastfeeding and exclusive breastfeeding rates both in Brazil and in this small city are better when compared to countries such as China and the United States^11^.

The 4.8 months increase observed between 2001 and 2013 was very similar to the rate of increase of 4.6 months observed in the 1996 to 2006 decade^4^. The median duration of breastfeeding found in the city also remained similar to that observed in Brazil in 2008 (11.3 months), although slightly above the median of the Southeast Region (10.1 months), and in the capital of the State of São Paulo (9.8 months)^7^.

In addition to the implementation of public policies to promote, protect and support breastfeeding, and the expansion of basic health care, the population profile may have influenced the breastfeeding indicators. The improvement in family income, an increase in the proportion of mothers who received counseling on breastfeeding during prenatal and childbirth care, and reduction in pacifier use may have contributed positively, as they have been shown to be associated with increased prevalence and duration of breastfeeding^8^ ^,^ ^10^ ^,^ ^17^, which highlights the relevance of nursing care in primary health care (the gateway to the health system), with the prioritization of maternal and child health care through appointments, educational groups, and home visits.

On the other hand, the increased proportion of mothers who were in the labor market and the decreased proportion of women with three years or more of education from 2001 to 2013 may have interfered negatively on the breastfeeding patterns observed in this study. Since there is evidence that mothers with better schooling present longer duration of breastfeeding^8^ and lower maternal schooling, as well as integration into work, are determinant for interruption of exclusive breastfeeding^17^, breastfeeding indicators could have been better if maternal profile was more favorable.

In 2013, the same correlates observed in 2001^12^ remained associated with the median duration of breastfeeding, confirming the pre-established hypothesis. Thus, the median duration of breastfeeding was lower among first-born children and among those who used pacifiers.

Birth order has been studied in terms of women’s parity and previous experience in breastfeeding. Primiparous women breastfeed for less time, either because of insecurity, younger age, less education, less knowledge about the benefits of breastfeeding, or less willingness to cope with social and cultural difficulties. However, it is necessary to consider that the intention of pregnant woman to breastfeed is another factor strongly related to the duration of the breastfeeding. In addition, each birth occurs in different family contexts, so the influence of this variable is difficult to analyze^20^. The approach to breastfeeding in the prenatal period, considering the situation of primiparous women, can reduce anxiety and even increase the number of pregnant women with the intention to breastfeed. On the other hand, the experience of breastfeeding from mothers of children born second or later in birth order must be valued, and can be considered as a protective factor for breastfeeding.

There is evidence that pacifier use is associated with maternal work outside the home, primiparity, and lack of breastfeeding within the first hour of birth^21^. Classically, pacifiers have been associated with lower frequency and duration of breastfeeding, especially exclusive breastfeeding^22^. Pacifier use has been discouraged since the beginning of the 2000s, however, it continues to be highly prevalent and influences negatively the maintenance of breastfeeding in our study, probably because it is a culturally accepted practice in Brazil, and also because the mechanisms involved in the relationship with weaning have not yet been fully elucidated^16^ ^,^ ^23^.

It is important to emphasize that the effectiveness of breastfeeding actions depends on the health professionals considering the complexity of the determinants of breastfeeding, and the life situation of the mothers^24^.

Thus, the results of this study increase the knowledge about the determinants of breastfeeding, in order that the actions to promote the practice have greater investments so that they have the desired impact, increase the prevalence and duration of breastfeeding and exclusive breastfeeding, and decrease the importance of factors such as birth order and pacifier use. In addition, this research can be easily replicated by primary care nurses to plan and evaluate actions to promote and support the practice of breastfeeding, which also demonstrated the importance of considering the specific characteristics in the care of each population. In our context, women with no prior experience in breastfeeding should be prioritized, and pacifier use needs to be discouraged.

Actions to promote breastfeeding, such as counseling on breastfeeding in prenatal and childbirth periods, breastfeeding within the first hour of life, exclusive breastfeeding in the maternity unit, childcare follow-up, and counseling during childcare appointments are reflections of the public policies of promotion and support for breastfeeding, implemented in the decade of 2000, so that is the reason why they were not evaluated in the 2001. In this context, one of the limitations of this study is the methodological difference in 2001 and 2013 designs. Although they are similar studies, they had different samples: the first was population-based; the second was performed with children enrolled in PHCFs, who attended the service for appointments. It could be expected that by 2013, the population would be subject to a bias, that is, that the actions of breastfeeding promotion would positively influence the duration of breastfeeding. However, the hypothesis that such actions would have a positive effect on the median duration of breastfeeding, in 2013, was not confirmed. Thus, it is necessary to reflect on the scope of the policies of promotion and support breastfeeding, which should be evaluated in other investigations on the operationalization of actions to promote the practice, not only in relation to the frequency, but also in the quality of these actions, which are performed in the health care services.

## Conclusions

There was an increase of 33.4% in the prevalence of exclusive breastfeeding, and 20.9% in breastfeeding. The median duration of breastfeeding increased from 7.2 to 12 months. In 2013, the same correlates identified in 2001 were also associated with a shorter duration of breastfeeding: being the first born, and pacifier use.
